# Supplementary material for: Modeling breast cancer progression to bone: how driver mutation order and metabolism matter
Source: BMC Med Genomics. 2019 Jul 25;12(Suppl 6):106. doi: 10.1186/s12920-019-0541-4 (PMC6657267; doi:10.1186/s12920-019-0541-4)
Supplement: Supplementary file 1 — Eigenstate representation of the cell state, accumulation of driver mutations in terms of ladder operators and method to retrieve the effective driver mutations. (PDF 209 kb) [file 12920_2019_541_MOESM1_ESM.pdf]

RESEARCH

Modeling breast cancer progression to bone: how driver mutation order and metabolism matter

Gianluca Ascolani[1,2*†] and Pietro Liò[1†]

*Correspondence: g.ascolani@sheffield.ac.uk
[1]Department of Computer Science and Technology, Computer Laboratory, University of Cambridge, William Gates Building, 15 JJ Thomson Avenue, CB3 0FD Cambridge, UK
Full list of author information is available at the end of the article
†Equal contributor

Additional File 1. Mutations and non-commuting operators

Additional File 1..1 Mutation operator and order of driver mutations

If we define the vector $g = \{g_1, \ldots, g_{\overline{m}_\mathrm{d}}\}$ where g_i is the i-th gene and the operator $\widehat{\mathrm{H}}_g$ such that given a set of genes S, $\widehat{\mathrm{H}}_g(S)$ returns a vector $\boldsymbol{\eta} = \{\eta_1, \ldots, \eta_{\overline{m}_\mathrm{d}}\}$ with components

$$\eta_i = \begin{cases} 1 & \text{if } \ g_i \in S \\ 0 & \text{otherwise} \end{cases}, \tag{1}$$

then applying $\widehat{\mathrm{H}}_g$ to each element of the sequence S' defined in Eq. (1) of the main text, we obtain

$$C = \widehat{\mathrm{H}}_g \circ S' = \{\widehat{\mathrm{H}}_g(S'_1), \ldots, \widehat{\mathrm{H}}_g(S'_{\overline{k}})\} = \{\boldsymbol{\eta}_1, \ldots, \boldsymbol{\eta}_{\overline{k}}\}.$$

C is the matrix in $[0,1]^{\overline{k} \times \overline{m}_\mathrm{d}}$ describing which mutated gene is considered necessary in a given compartment where the rows $\boldsymbol{\eta}_k$ represent the genes and the columns C_j identify a gene in the compartments. One or more genes may be important in various compartments and C may not be a full rank matrix.

Let us define a state vector $\boldsymbol{m}_j \in \mathbb{R}^2$ corresponding to the gene g_j in the mutations representation so that, in the standard basis $\mathcal{B}_{m_j} = \left\{ \binom{1}{0} \ \binom{0}{1} \right\}$, we can introduce the mutation observable as a diagonal matrix operator

$$\widehat{M}_j = \begin{pmatrix} 1 & 0 \\ 0 & -1 \end{pmatrix}$$

which acts on the respective eigenvectors as follows: $\widehat{M}_j\left(\boldsymbol{m}_j\right) = \lambda_{m_j} \boldsymbol{m}_j$. The mutation operator has two eigenvalues λ_{m_j}: 1 and -1 representing the case gene g_j is mutated or not mutated, respectively. The corresponding eigenvectors are equal to the basis vectors. Generally, there are multiple genes involved in the dysfunction of a cell or in the appearance of a new oncogenic behaviour. Hence, the vector space of the genes' multitude can be represented as the direct product of single gene vectors:

$$\boldsymbol{m} = \bigotimes_{j=1}^{\overline{m}_\mathrm{d}} \boldsymbol{m}_j \in \mathcal{M} \subseteq \mathbb{R}^{\left(2^{\overline{m}_\mathrm{d}}\right)},$$

and, similarly, the mutation operator for multiple genes is given by:

$$\widehat{M} = \bigotimes_{j=1}^{\overline{m_{\mathrm{d}}}} \widehat{M}_j,$$

where, formally, the symbol $\otimes$ can be interpreted as the Kronecker product of the operators $\widehat{M}_j$. The mutations operator applied to an eigenvector of genes returns the eigenvector times the product of all the genes' eigenstates:

$$\widehat{M}\left(\boldsymbol{m}\right) = \prod_{j=1}^{\overline{m_{\mathrm{d}}}} \lambda_{m_j} \boldsymbol{m} = \lambda_M \boldsymbol{m}.$$

From the mutation operator $\widehat{M}_j$ and the elements c_{kj} of the matrix C of necessary genes in a compartment, we derive the operator for a single gene:

$$\widehat{D}_{kj} = \widehat{\mathbb{I}} - \frac{1}{2} c_{kj} \left(\widehat{\mathbb{I}} - \widehat{M}_j\right) = \begin{pmatrix} 1 & 0 \\ 0 & 1 - c_{kj} \end{pmatrix}$$

which measures the deregulation introduced by a gene in a set of important alterable genes like those belonging to a genetic pathway. $\widehat{D}_{kj}$ is diagonal in the mutations representation ($\mathcal{B}_{m_j}$), and the two eigenvalues $\lambda_{D_{kj}}$ are degenerate with value 1 if the gene g_j is unimportant in compartment k, while they are non degenerate if the gene is important. In the latter case, $\lambda_{D_{kj}}$ is 0 when g_j mutated and 1 otherwise. As shown before, we can use the direct product over all the genes to define the deregulated pathway operator referring to compartment k (or to a genetic pathway) for all the involved genes:

$$\widehat{D}_k = \bigotimes_{j=1}^{\overline{m_{\mathrm{d}}}} \widehat{D}_{kj}.$$

If the state vector $\boldsymbol{m}$ is an eigenvector of $\widehat{M}$, it is also an eigenvector of the deregulated pathway operator:

$$\widehat{D}_k\left(\boldsymbol{m}\right) = \prod_{j=1}^{\overline{m_{\mathrm{d}}}} \lambda_{D_{kj}} \boldsymbol{m} = \lambda_{D_k} \boldsymbol{m},$$

and the eigenvalue $\lambda_{D_k} \in \{0, 1\}$ determines if all the genes in compartment k are mutated. In case we want to compare the state of two different cells or the states of a cell at two different times given by $\boldsymbol{m1}$ and $\boldsymbol{m2}$, we define the inner product $\langle \boldsymbol{m2}, \boldsymbol{m1} \rangle = \delta_{m1,m2}$ so that:

$$\left\langle \boldsymbol{m2}, \widehat{D}_k\left(\boldsymbol{m1}\right) \right\rangle = \lambda_{D_k}\ \delta_{m1,m2}.$$

It is straightforward noticing that the deregulated pathway operator allows us to properly address the problem of inter compartmental unordered cumulated mutations and the intra-compartmental order of mutation through the index k just by imposing the orthogonality between elements belonging to different compartments.

In fact, if $k1$ and $k2$ are two generic compartments, and $\boldsymbol{m}_{k1}$ and $\boldsymbol{m}_{k2}$ are the mutation vectors referring to the multiple genes in the respective compartments, then:

$$\widehat{D}_{k1}\left(\boldsymbol{m}_{k2}\right)=\begin{cases}\lambda_{D_{k1}}\boldsymbol{m}_{k1} & \text{k1=k2}\\ 0 & \text{otherwise}\end{cases}.$$

To easily deal with the order of mutation among compartments in terms of operators, we need to extend the notation by defining the level state vector $\boldsymbol{\ell}\in\mathcal{L}$ where the vector space $\mathcal{L}$ is given by the direct sum of $\overline{k}$ identical $\mathcal{M}$ space vector, which for convenience we rename as $\mathcal{M}_k$, such that:

$$\mathcal{L}=\bigoplus_{k=1}^{\overline{k}}\mathcal{M}=\bigoplus_{k=1}^{\overline{k}}\mathcal{M}_k\subseteq\mathbb{R}^{\left(2^{\overline{m_\mathrm{d}}}\right)\cdot\overline{k}}.$$

An opportune basis for $\mathcal{L}$ is the standard basis where the first $2^{\overline{m_\mathrm{d}}}$ basis vectors are a complete basis for $\mathcal{M}_1$, and their linear combinations refer to the mutation states of all the genes while a cell is in compartment $k=1$. The basis vectors having the only component different from zero at positions going from $2^{\overline{m_\mathrm{d}}}+1$ to $2^{\overline{m_\mathrm{d}}+1}$ are a complete basis for $\mathcal{M}_2$, and their linear combinations refer to the mutation state of all the genes while the cell is in compartment $k=2$. The partition of the basis vectors of $\mathcal{L}$ can be similarly done for each compartment.

Let us call $\boldsymbol{\ell}_k$ the gene state eigenvectors of a cell in compartment k such that the components from $2^{\overline{m_\mathrm{d}}+k}+1$ to $2^{\overline{m_\mathrm{d}}+k+1}$ are identical to the respective eigenvectors $\boldsymbol{m}_k\in\mathcal{M}_k$ in the k-th compartment. In this representation, we can redefine and unify the set of deregulated pathway operators in a simpler form:

$$\widehat{D}=\begin{pmatrix}\widehat{D}_1 & 0 & \cdots & 0\\ 0 & \widehat{D}_2 & \cdots & 0\\ \vdots & \vdots & \ddots & 0\\ 0 & 0 & \cdots & \widehat{D}_{\overline{k}}\end{pmatrix},$$

and $\widehat{D}\left(\boldsymbol{\ell}_k\right)=\lambda_{D_k}\boldsymbol{\ell}_k$.

It is important to stress all the state vectors defined above are all factorizable because genes and compartments are all considered independent.

Additional File 1..2 Ladder operators and effective driver mutations

The deregulated pathways operator $\widehat{D}$ is diagonal in the representation of the orthonormal level basis $\mathcal{B}_{\boldsymbol{\ell}_k}=\{\boldsymbol{\ell}_1,\boldsymbol{\ell}_2,\ldots,\boldsymbol{\ell}_{\overline{k}}\}$, and it only has two degenerate eigenvalues λ_D equal to 0 and 1; the null eigenvalue means not all the required genes involved in the regulation/compensation of a specific pathway are mutated so as to not result in further oncogenic activity, and the latter eigenvalue means the pro-oncogenic behavior is present and the cell can use it when needed. Let us introduce the ladder operators for the level of the path $\widehat{L}^+$ and $\widehat{L}^-$. The two operators act on the basis by increasing or decreasing the level respectively. Therefore, the creation operator gives $\widehat{L}^+\left(\boldsymbol{\ell}_k\right)=\boldsymbol{\ell}_{k+1}$ for any integer $1\leq k<\overline{k}$, and it returns 0

when applied on $\boldsymbol{\ell}_{\overline{k}}$. In the opposite way, the action of the annihilation operator is $\widehat{L}^{-}(\boldsymbol{\ell}_k) = \boldsymbol{\ell}_{k-1}$ for any integer $1 < k \leq \overline{k}$, and it gives 0 when applied on $\boldsymbol{\ell}_1$. The ladder operators are the transpose of one another: $\widehat{L}^{+} = \left(\widehat{L}^{-}\right)^{\mathrm{T}}$. For extended discussions on ladder operators and their applications see [1, 2, 3],

Coupling the degenerate operator and the ladder operators such that $\widehat{B}^{+} = \widehat{L}^{+}\widehat{D}$ and $\widehat{B}^{-} = \widehat{D}\widehat{L}^{-}$ is useful to determine which one of a succession of ordered events in the cell path represents a barrier. The barrier operators $\widehat{B}^{+}$ and $\widehat{B}^{-}$ applied to the elements of the basis vectors $\mathcal{B}_{\boldsymbol{\ell}_k}$ give

$$\widehat{B}^{+}(\boldsymbol{\ell}_k) = \lambda_{D_k}\widehat{L}^{+}(\boldsymbol{\ell}_k) = \lambda_{D_k}\boldsymbol{\ell}_{k+1}$$

and

$$\widehat{B}^{-}(\boldsymbol{\ell}_k) = \widehat{D}(\boldsymbol{\ell}_{k-1}) = \lambda_{D_{k-1}}\boldsymbol{\ell}_{k-1}$$

if $1 \leq k+1 \leq \overline{k}$ and zero otherwise.

In order to determine the value of the transition rate of each cell to jump from one compartment to the next, it is necessary to determine how many driver mutations are effective for the specific cell. In a system with a finite number of levels, meaning a finite number of compartments, this computation can be achieved by using the effective ladder operators $\widehat{E}^{\pm} = \frac{1}{1-\widehat{B}^{\pm}}$.

The result of the effective ladder operators on the basis vector $\boldsymbol{\ell}_k$ is:

$$\widehat{E}^{\pm}(\boldsymbol{\ell}_k) = \sum_{\substack{j=0 \\ 1\leq k\pm j\leq \overline{k}}} \left(\prod_{i=0}^{j} \lambda_{D\,k\pm i}\right) \boldsymbol{\ell}_{k\pm j}$$

where the factors $\left(\prod_{i=0}^{j} \lambda_{D\,k\pm i}\right)$ are different from zero only when all the ordered sequence of driver mutations previous to (in case of $\widehat{E}^{-}$) and afterwards (in case of $\widehat{E}^{+}$) the unknown value m_{e} in a given compartment k have already been acquired. The number of effective driver mutations m_{e} for a cell in compartment k is finally retrieved by the square of the norm of $\widehat{E}^{-}(\boldsymbol{\ell}_k)$ as follows:

$$\left\langle \boldsymbol{\ell_k}, \widehat{E}^{+}\widehat{E}^{-}(\boldsymbol{\ell}_k)\right\rangle = \sum_{\substack{j=0 \\ 1\leq k\pm j\leq \overline{k}}} \left(\prod_{i=0}^{j} \lambda_{D\,k\pm i}\right) = m_{\mathrm{e}}. \tag{2}$$

This result can be applied to find the value of r_{pass} of each cell in each compartment and at each simulation step by knowing the cell mutated genes.

Author details
[1]Department of Computer Science and Technology, Computer Laboratory, University of Cambridge, William Gates Building, 15 JJ Thomson Avenue, CB3 0FD Cambridge, UK. [2]Department of Oncology & Metabolism, The University of Sheffield, Medical School, Beech Hill Road, S10 2RX Sheffield, UK.

References

1. Aleksandra M. Walczak, Andrew Mugler, and Chris H. Wiggins. *Analytic Methods for Modeling Stochastic Regulatory Networks*, pages 273–322. Humana Press, Totowa, NJ, 2012.
2. J.J. Sakurai and J. Napolitano. *Modern Quantum Mechanics*. Pearson new international edition. Pearson, 2013.
3. E. Zeidler. *Quantum Field Theory I: Basics in Mathematics and Physics: A Bridge between Mathematicians and Physicists*. Quantum Field Theory. Springer Berlin Heidelberg, 2007.
