# Supplementary material for: Modeling breast cancer progression to bone: how driver mutation order and metabolism matter
Source: BMC Med Genomics. 2019 Jul 25;12(Suppl 6):106. doi: 10.1186/s12920-019-0541-4 (PMC6657267; doi:10.1186/s12920-019-0541-4)
Supplement: Supplementary file 2 — Mathematical derivation of the master equation for a birth and death process subordinated to the occurrence of metabolic mutations affecting the cellular proliferation rate and subjected to the order of driver mutations and to non-driver mutations. (PDF 181 kb) [file 12920_2019_541_MOESM2_ESM.pdf]

RESEARCH

Modeling breast cancer progression to bone: how driver mutation order and metabolism matter

Gianluca Ascolani[1,2*†] and Pietro Liò[1†]

*Correspondence: g.ascolani@sheffield.ac.uk
[1]Department of Computer Science and Technology, Computer Laboratory, University of Cambridge, William Gates Building, 15 JJ Thomson Avenue, CB3 0FD Cambridge, UK
Full list of author information is available at the end of the article
†Equal contributor

Additional File 2. Master equation subordinated to a mutation process

For the sake of consistency, let us rewrite here the conditional probability density in the natural time and the meaning of each term as expressed in the main text.

The conditional probability density $\rho(\boldsymbol{d}, m_{\rm n}, m_{\rm m})$ that a cell starting from the state $\sigma_0 = \{\boldsymbol{0}, 0, 0, k\}$ arrives after $m_{\rm m}$ steps to the state $\sigma = \{\boldsymbol{d}, m_{\rm n}, m_{\rm m}, k\}$ is:

$$
\begin{aligned}
\rho(\boldsymbol{d}, m_{\rm n}, m_{\rm m}, k) \; = \;& \\
\Bigg[\frac{1}{\overline{m}_{\rm d}} \sum_{i=1}^{\overline{m}_{\rm d}} & r_{\rm asym}(m_{\rm d}-1, m_{\rm n}, k) r_{\rm d}(m_{\rm d}-1, m_{\rm n}, k) \rho(\boldsymbol{d}-\boldsymbol{u_i}, m_{\rm n}, m_{\rm m}-1, k) \\
& + r_{\rm asym}(m_{\rm d}, m_{\rm n}-1, k)\; r_{\rm n}(m_{\rm d}, m_{\rm n}-1, k)\; \rho(\boldsymbol{d}, m_{\rm n}-1, m_{\rm m}-1, k)\Bigg] \\
& + 2\Bigg[\frac{1}{\overline{m}_{\rm d}} \sum_{i=1}^{\overline{m}_{\rm d}} r_{\rm sym}(m_{\rm d}-1, m_{\rm n}, k)\; r_{\rm d}(m_{\rm d}-1, m_{\rm n}, k)\; \rho(\boldsymbol{d}-\boldsymbol{u_i}, m_{\rm n}, m_{\rm m}-1, k) \\
& + r_{\rm sym}(m_{\rm d}, m_{\rm n}-1, k)\; r_{\rm n}(m_{\rm d}, m_{\rm n}-1, k)\; \rho(\boldsymbol{d}, m_{\rm n}-1, m_{\rm m}-1, k)\Bigg] \\
& - r_{\rm sym}(m_{\rm d}, m_{\rm n}, k)\; \rho(\boldsymbol{d}, m_{\rm n}, m_{\rm m}, k) - r_{\rm apop}(m_{\rm d}, m_{\rm n}, k)\rho(\boldsymbol{d}, m_{\rm n}, m_{\rm m}, k) \\
& + [r_{\rm pass}(m_{\rm d}, m_{\rm n}, k-1)\rho(\boldsymbol{d}, m_{\rm n}, m_{\rm m}, k-1) - r_{\rm pass}(m_{\rm d}, m_{\rm n}, k)\rho(\boldsymbol{d}, m_{\rm n}, m_{\rm m}, k)]
\end{aligned}
\tag{1}
$$

The first term enclosed in square brackets describes the increase of number of cells in the state $\sigma = \{\boldsymbol{d}, m_{\rm n}, m_{\rm m}, k\}$ due to asymmetric proliferation. The mutation occurring during the asymmetric proliferation can be driver or passenger; hence $r_d(\sigma) + r_n(\sigma) = 1$. The second term enclosed in square brackets takes into account the increase of cells due to symmetric proliferation, while the third term express the fact that both the daughter cells equally change their state and there is no self-renewal. The fourth term is the decreasing of cells due to apoptosis, and the last term is due to the change of compartment.

In order to compare the dynamics with the biological process, we can switch from the natural time of the events to the physical time [1], meaning the time is measured with a macroscopic clock advancing with a regular periodic step. To do so, we introduce the conditional probability density $\rho(\boldsymbol{d}, m_{\rm n}, m_{\rm m}, k, t)$ that exactly at time t a cell, starting a time $t = t_0$ in the state $\sigma_0 = \{\boldsymbol{0}, 0, 0, k\}$, changes its state to $\sigma = \{\boldsymbol{d}, m_{\rm n}, m_{\rm m}, k\}$:

$$\rho(\boldsymbol{d}, m_{\mathrm{n}}, m_{\mathrm{m}}, k, t) = \int_0^t \Bigg\{ \psi(m_{\mathrm{m}}-1, t-t') \tag{2}$$

$$\Bigg[\Bigg(\frac{1}{\overline{m_{\mathrm{d}}}}\sum_{i=1}^{\overline{m_{\mathrm{d}}}} r_{\mathrm{asym}}\, r_{\mathrm{d}}\, \rho(\boldsymbol{d}-\boldsymbol{u_i}, m_{\mathrm{n}}, m_{\mathrm{m}}-1, k, t') + r_{\mathrm{asym}}\, r_{\mathrm{n}} \rho(\boldsymbol{d}, m_{\mathrm{n}}-1, m_{\mathrm{m}}-1, k, t')\Bigg)$$

$$+2\Bigg(\frac{1}{\overline{m_{\mathrm{d}}}}\sum_{i=1}^{\overline{m_{\mathrm{d}}}} r_{\mathrm{sym}}\, r_{\mathrm{d}}\, \rho(\boldsymbol{d}-\boldsymbol{u_i}, m_{\mathrm{n}}, m_{\mathrm{m}}-1, k, t') + r_{\mathrm{sym}}\, r_{\mathrm{n}}\, \rho(\boldsymbol{d}, m_{\mathrm{n}}-1, m_{\mathrm{m}}-1, k, t')\Bigg)\Bigg]$$

$$+\, \psi(m_{\mathrm{m}}, t-t') \Bigg[- r_{\mathrm{sym}}\; \rho(\boldsymbol{d}, m_{\mathrm{n}}, m_{\mathrm{m}}, k, t') - r_{\mathrm{apop}}\; \rho(\boldsymbol{d}, m_{\mathrm{n}}, m_{\mathrm{m}}, k, t')$$

$$+ \Bigg(r_{\mathrm{pass}}\; \rho(\boldsymbol{d}, m_{\mathrm{n}}, m_{\mathrm{m}}, k-1, t') - r_{\mathrm{pass}}\; \rho(\boldsymbol{d}, m_{\mathrm{n}}, m_{\mathrm{m}}, k, t')\Bigg)\Bigg]\Bigg\}\ \mathrm{d}t',$$

where $\psi(i,t)$ is the waiting time distribution that the i-th mutational event occurs exactly at time t, and the corresponding survival probability is given by the relation $\Psi(i,t) = 1 - \int_0^t \psi(i,t')\ \mathrm{d}t'$. The integral in Eq. 2 means that the event before the last may have occurred at any possible time t' between 0 and t.

Let us consider an ensemble of cells, whose genome generates a trajectory in the mutation state which starts from a common initial state (the healthy state) and evolves in time. If we let the system run and then freeze it, what we see is a population of cells which are in different states. Differently from the natural time frame, we observe cells which have accomplished unequal amounts of jumps; therefore, if i is associated to the age of the cells, then at any time i in the natural time frame, the system is composed of cells having the same age, while at any instant t in the physical time frame, there are cells with few mutations together with cells which have accumulated a large number of mutations.

The total amount of cells with $\sigma = \{\boldsymbol{d}, m_{\mathrm{n}}, m_{\mathrm{m}}, k\}$ mutations observed at time t are the sum of all cells which changed their state to σ (with any possible order of the occurrence of $\boldsymbol{d}$ and n) at any earlier time t' without further events between the time t' and t included those jumping exactly at time t. Hence the probability density $p(\boldsymbol{d}, m_{\mathrm{n}}, m_{\mathrm{m}}, k, t)$ of finding a cell in σ is:

$$\begin{aligned} p(\boldsymbol{d}, m_{\mathrm{n}}, m_{\mathrm{m}}, k, t) &= \int_0^t \rho(\boldsymbol{d}, m_{\mathrm{n}}, m_{\mathrm{m}}, k, t')\ \Psi(m_{\mathrm{m}}, t-t')\ \mathrm{d}t' \\ &= \delta(\boldsymbol{d}-\boldsymbol{d}_0)\delta(m_{\mathrm{n}}-n_0)\Psi(m_{\mathrm{m}}, t) \\ &\quad + \int_0^t \rho^+(\boldsymbol{d}, m_{\mathrm{n}}, m_{\mathrm{m}}, k, t')\Psi(m_{\mathrm{m}}, t-t')\ \mathrm{d}t', \end{aligned} \tag{3}$$

where we have performed a Riemann-Stieltjes integral over time with a discontinuity of the conditional probability densities ρ in $t=0$ which can be expressed as:

$$\rho(\boldsymbol{d}, m_{\mathrm{n}}, m_{\mathrm{m}}, k, t') = \delta(m_{\mathrm{n}}-n_0)\delta(t-0^+) + \rho^+(\boldsymbol{d}, m_{\mathrm{n}}, m_{\mathrm{m}}, k, t'). \tag{4}$$

The time derivative of the previous equation give:

$$\begin{aligned}
&\frac{\mathrm{d}}{\mathrm{d}t}\, p(\boldsymbol{d}, m_{\mathrm{n}}, m_{\mathrm{m}}, k, t) \\
&\quad = \rho^{+}(\boldsymbol{d}, m_{\mathrm{n}}, m_{\mathrm{m}}, k, t) \\
&\qquad - \int_0^t \rho^{+}(\boldsymbol{d}, m_{\mathrm{n}}, m_{\mathrm{m}}, k, t')\psi(m_{\mathrm{m}}, t-t')\,\mathrm{d}t' - \delta(\boldsymbol{d}-\boldsymbol{d}_0)\psi(m_{\mathrm{m}}, t)
\end{aligned} \tag{5}$$

where the flux of particle exiting the σ state is:

$$\begin{aligned}
j(\boldsymbol{d}, m_{\mathrm{n}}, m_{\mathrm{m}}, k, t) &= \int_0^t \rho(\boldsymbol{d}, m_{\mathrm{n}}, m_{\mathrm{m}}, k, t')\psi(m_{\mathrm{m}}, t-t')\,\mathrm{d}t' \\
&= \int_0^t \rho^{+}(\boldsymbol{d}, m_{\mathrm{n}}, m_{\mathrm{m}}, k, t')\psi(m_{\mathrm{m}}, t-t')\,\mathrm{d}t' + \delta(\boldsymbol{d}) - \boldsymbol{d}_0)\psi(m_{\mathrm{m}}, t).
\end{aligned} \tag{6}$$

Therefore, the master equation in terms of incoming and outgoing fluxes are:

$$\frac{\mathrm{d}}{\mathrm{d}t}\, p(\boldsymbol{d}, m_{\mathrm{n}}, m_{\mathrm{m}}, k, t) = \rho^{+}(\boldsymbol{d}, m_{\mathrm{n}}, m_{\mathrm{m}}, k, t) - j(\boldsymbol{d}, m_{\mathrm{n}}, m_{\mathrm{m}}, k, t). \tag{7}$$

Using Eq. 4, we can write the incoming flux in terms of the outgoing flux defined in Eq. 6. The explicit result for the specific set of reaction is:

$$\begin{aligned}
&\rho^{+}(\boldsymbol{d}, m_{\mathrm{n}}, m_{\mathrm{m}}, k, t) = \Bigg\{ \\
&\quad \left[\frac{1}{\overline{m_{\mathrm{d}}}} \sum_{i=1}^{\overline{m_{\mathrm{d}}}} r_{\mathrm{asym}}\ r_{\mathrm{d}}\ j(\boldsymbol{d}-\boldsymbol{u_i}, m_{\mathrm{n}}, m_{\mathrm{m}}, k, t) + r_{\mathrm{asym}}\ r_{\mathrm{n}} j(\boldsymbol{d}, m_{\mathrm{n}}-1, m_{\mathrm{m}}, k, t)\right] \\
&\quad +2\left[\frac{1}{\overline{m_{\mathrm{d}}}} \sum_{i=1}^{\overline{m_{\mathrm{d}}}} r_{\mathrm{sym}}\ r_{\mathrm{d}}\ j(\boldsymbol{d}-\boldsymbol{u_i}, m_{\mathrm{m}}, k, t) + r_{\mathrm{sym}}\ r_{\mathrm{n}}\ j(\boldsymbol{d}, m_{\mathrm{n}}-1, m_{\mathrm{m}}, k, t)\right] \\
&\qquad - r_{\mathrm{sym}}\ j(\boldsymbol{d}, m_{\mathrm{n}}, m_{\mathrm{m}}, k, t) - r_{\mathrm{apop}}\ j(\boldsymbol{d}, m_{\mathrm{n}}, m_{\mathrm{m}}, k, t) \\
&\qquad + \left[r_{\mathrm{pass}}\ j(\boldsymbol{d}, m_{\mathrm{n}}, m_{\mathrm{m}}, k-1, t) - r_{\mathrm{pass}}\ j(\boldsymbol{d}, m_{\mathrm{n}}, m_{\mathrm{m}}, k, t)\right]\Bigg\}.
\end{aligned}$$

In order to express the master equation only in terms of the probability density (see the derivation in [2] for more general case), we can Laplace transform both Eq. 3 and Eq. 6:

$$\mathcal{L}\{p(\boldsymbol{d}, m_{\mathrm{n}}, m_{\mathrm{m}}, k, t)\} = \widetilde{p}(\boldsymbol{d}, m_{\mathrm{n}}, m_{\mathrm{m}}, k, s) = \mathcal{L}\{\rho(\boldsymbol{d}, m_{\mathrm{n}}, m_{\mathrm{m}}, k, t)\}\ \mathcal{L}\{\Psi(m_{\mathrm{m}}, t)\},$$

$$\mathcal{L}\{j(\boldsymbol{d}, m_{\mathrm{n}}, m_{\mathrm{m}}, k, t)\} = \widetilde{j}(\boldsymbol{d}, m_{\mathrm{n}}, m_{\mathrm{m}}, k, s) = \mathcal{L}\{\rho(\boldsymbol{d}, m_{\mathrm{n}}, m_{\mathrm{m}}, k, t)\}\ \mathcal{L}\{\psi(m_{\mathrm{m}}, t)\},$$

from which it is easy to derive the relation between the transformed probability density $\widetilde{p}$ and the transformed influx of cells $\widetilde{j}$ in terms of the transformed memory kernel $\widetilde{K}$:

$$\widetilde{j}(\boldsymbol{d}, m_{\mathrm{n}}, m_{\mathrm{m}}, k, s) = \widetilde{p}(\boldsymbol{d}, m_{\mathrm{n}}, m_{\mathrm{m}}, k, s)\frac{\widetilde{\psi}(m_{\mathrm{m}}, s)}{\widetilde{\Psi}(m_{\mathrm{m}}, s)}, \tag{8}$$

and

$$\widetilde{K}(m_{\mathrm{m}}, s) = \frac{\widetilde{\psi}(m_{\mathrm{m}}, s)}{\widetilde{\Psi}(m_{\mathrm{m}}, s)}. \tag{9}$$

The previous results allow us to rewrite the master equation in terms only of the Laplace transform of the probability density:

$$\begin{aligned}
&\mathcal{L}\left\{\frac{\mathrm{d}}{\mathrm{d}t}\, p(\boldsymbol{d}, m_{\mathrm{n}}, m_{\mathrm{m}}, k, t)\right\} = s\ \widetilde{p}(\boldsymbol{d}, m_{\mathrm{n}}, m_{\mathrm{m}}, k, s) - p(\boldsymbol{d}, m_{\mathrm{n}}, m_{\mathrm{m}}, k, 0) \qquad (10)\\
&= \widetilde{K}(m_{\mathrm{m}} - 1, s)\\
&\left\{\left[\frac{1}{\overline{m_{\mathrm{d}}}} \sum_{i=1}^{\overline{m_{\mathrm{d}}}} r_{\mathrm{asym}}\ r_{\mathrm{d}}\ \widetilde{p}(\boldsymbol{d} - \boldsymbol{u_i}, m_{\mathrm{n}}, m_{\mathrm{m}} - 1, k, s) + r_{\mathrm{asym}}\ r_{\mathrm{n}}\ \widetilde{p}(\boldsymbol{d}, m_{\mathrm{n}}, m_{\mathrm{m}} - 1, k, s)\right]\right.\\
&\left.+ 2\left[\frac{1}{\overline{m_{\mathrm{d}}}} \sum_{i=1}^{m_{\mathrm{m}}} r_{\mathrm{sym}}\ r_{\mathrm{d}}\ \widetilde{p}(\boldsymbol{d} - \boldsymbol{u_i}, m_{\mathrm{n}}, m_{\mathrm{m}} - 1, k, s) + r_{\mathrm{sym}}\ r_{\mathrm{n}}\ \widetilde{p}(\boldsymbol{d}, m_{\mathrm{n}}, m_{\mathrm{m}} - 1, k, s)\right]\right\}\\
&\quad + \widetilde{K}(m_{\mathrm{m}}, s)\left\{ - r_{\mathrm{sym}}\ \widetilde{p}(\boldsymbol{d}, m_{\mathrm{n}}, m_{\mathrm{m}}, k, s) - r_{\mathrm{apop}}\ \widetilde{p}(\boldsymbol{d}, m_{\mathrm{n}}, m_{\mathrm{m}}, k, s)\right.\\
&\qquad\qquad \left. + \left[r_{\mathrm{pass}}\ \widetilde{p}(\boldsymbol{d}, m_{\mathrm{n}}, m_{\mathrm{m}}, k - 1, s) - r_{\mathrm{pass}}\ \widetilde{p}(\boldsymbol{d}, m_{\mathrm{n}}, m_{\mathrm{m}}, k, s)\right]\right\},
\end{aligned}$$

and the Laplace transform of the memory kernel

$$\widetilde{K}(m_{\mathrm{m}}, s) = \frac{\widetilde{\psi}(m_{\mathrm{m}}, s)}{\widetilde{\Psi}(m_{\mathrm{m}}, s)}. \tag{11}$$

The master equation can be directly anti-Laplace transformed resulting in integro-differential equations with memory kernel $K(m_{\mathrm{m}}, t)$. Nevertheless, explicitly introducing the functional form of the waiting time distributions may result in further simplifications.

Author details
[1]Department of Computer Science and Technology, Computer Laboratory, University of Cambridge, William Gates Building, 15 JJ Thomson Avenue, CB3 0FD Cambridge, UK. [2]Department of Oncology & Metabolism, The University of Sheffield, Medical School, Beech Hill Road, S10 2RX Sheffield, UK.

References

1. B. I. Henry, T. A. M. Langlands, and S. L. Wearne. Anomalous diffusion with linear reaction dynamics: From continuous time random walks to fractional reaction-diffusion equations. *Phys. Rev. E*, 74:031116, Sep 2006.
2. Sergei Fedotov. Non-markovian random walks and nonlinear reactions: Subdiffusion and propagating fronts. *Phys. Rev. E*, 81:011117, Jan 2010.
